# Resveratrol Inhibits KSHV Reactivation by Lowering the Levels of Cellular EGR-1

**DOI:** 10.1371/journal.pone.0033364

**Published:** 2012-03-12

**Authors:** Ossie F. Dyson, Lia R. Walker, Adrian Whitehouse, Paul P. Cook, Shaw M. Akula

**Affiliations:** 1 Department of Microbiology and Immunology, Brody School of Medicine, East Carolina University, Greenville, North Carolina, United States of America; 2 Department of Internal Medicine, Brody School of Medicine, East Carolina University, Greenville, North Carolina, United States of America; 3 Faculty of Biological Sciences, Institute of Molecular and Cellular Biology, University of Leeds, Leeds, United Kingdom; University of Hong Kong, Hong Kong

## Abstract

In the field of herpesvirus research, the exact molecular mechanism by which such viruses reactivate from latency remains elusive. Kaposi's sarcoma-associated herpesvirus (KSHV) primarily exists in a latent state, while only 1–3% of cells support lytic infection at any specific time. KSHV reactivation from latency is an exceedingly intricate process mediated by the integration of viral and cellular factors. Previously, our lab has described early growth response-1 (Egr-1) as an essential component for the KSHV reactivation process via its ability to mediate transcription of KSHV *ORF50*, the gene encoding for replication and transcription activator (RTA), a viral component known to control the switch from latent to lytic infection. In here, electrophoretic mobility shift assays (EMSA) and chromatin immunoprecipitation (ChIP) experiments revealed that Egr-1 binds KSHV *ORF50* promoter (*ORF50*P) in at least two different GC-rich binding domains. Expression profiles of cellular *egr-1* and KSHV-encoded *ORF50* follow a similar pattern during *de novo* KSHV infection. Over-expressing Egr-1, a signaling component downstream of Raf>MEK>ERK1/2, in KSHV-infected cells activates KSHV lytic replication. Through performing more physiologically relevant experiments, we analyzed the effect of a dietary supplement containing resveratrol on KSHV-infected cells. Our results, for the first time, demonstrate resveratrol to act in lowering ERK1/2 activity and expression of Egr-1 in KSHV-infected cells, resulting in the suppression of virus reactivation from latency. Taken together, these findings will undoubtedly contribute to future studies on not only combating KSHV related disease conditions, but also on other herpesviruses-induced pathogenesis.

## Introduction

Significant strides have been made since the discovery of Kaposi's sarcoma-associated herpesvirus (KSHV) by Chang et al [1] nearly 20 years ago that have helped to increase our understanding of this infectious agent. KSHV is a γ2-herpesvirus that has been directly linked to the development of Kaposi's sarcoma (KS), primary effusion (PEL), and multicentric Castleman disease (MCD). This virus is closely related to Epstein-Barr virus (EBV), murine gammaherpesvirus-68, and herpesvirus saimiri [2]. The prevalence of KSHV infection varies depending on the geographical location with highest levels observed in Africa, where it has been reported to be greater than 40% [3]. As KSHV displays several characteristics shared among other herpesviruses, its ability to switch between latent and lytic stages of infection is of particular concern. The virus remains predominantly in a latent state, while 1–3% of cells may support a lytic infection at any given time [4]. Regulation of the switch between the two stages of infection is mediated by viral and cellular factors. Specifically, the KSHV protein, replication and transcription activator (RTA), is known to be a crucial viral component controlling the transition from latency to a lytic infection [5]. Recently, cellular early growth response-1 (Egr-1) protein was also shown to be an important factor involved in KSHV reactivation through its ability to mediate transcription of KSHV *ORF50*, the gene encoding RTA [6].

Egr-1 is a transcription factor that is also known as zif268, Krox-24, NGFI-A, and TIS8 [7]. It is induced by several external stimuli including growth factors, different forms of stress, and hormones. As a result of stimulation from various factors, e*gr-1* gene products advance to play a role in several cellular functions such as, but not limited to, growth, proliferation, and differentiation [8]. Egr-1 is part of a zinc-finger gene family that includes Egr-2, Egr-3, Egr-4, and the Wilms tumor suppressor (WT1) [9]. TPA is used to activate a lytic infection in KSHV-infected cells [10]. Egr-1 mediates the effect of TPA activation and is a downstream target of MAPK signaling [9], [11]. Furthermore, MAPK signaling is crucial for triggering KSHV reactivation from latency [12], [13]. However, despite the ability of Egr-1 and KSHV *ORF50* to interact with each other, there is little information available describing this association.

In a recent study, the ability of Egr-1 to bind KSHV *ORF50* promoter (*ORF50*P) was described [6]. In this report, electrophoretic mobility shift assays (EMSA) and chromatin immunoprecipitation (ChIP) experiments were employed to determine the locations on *ORF50*P that have an affinity for Egr-1 binding. Our results demonstrated at least two targets that are likely crucial for mediating Egr-1 binding to *ORF50*P. These findings were confirmed through the use of mutation studies. In addition, we tested the ability of resveratrol, a naturally occurring product found in a variety of fruits and nuts [14], in regulating MAPK signaling>Egr-1 expression>promoting virus latency. As such, the ramifications on the ability of Egr-1-induced transcription of *ORF50* in viral pathogenesis are discussed.

## Results

### Egr-1 binds at least two different sites within the *ORF50*P

**Figure 1:**
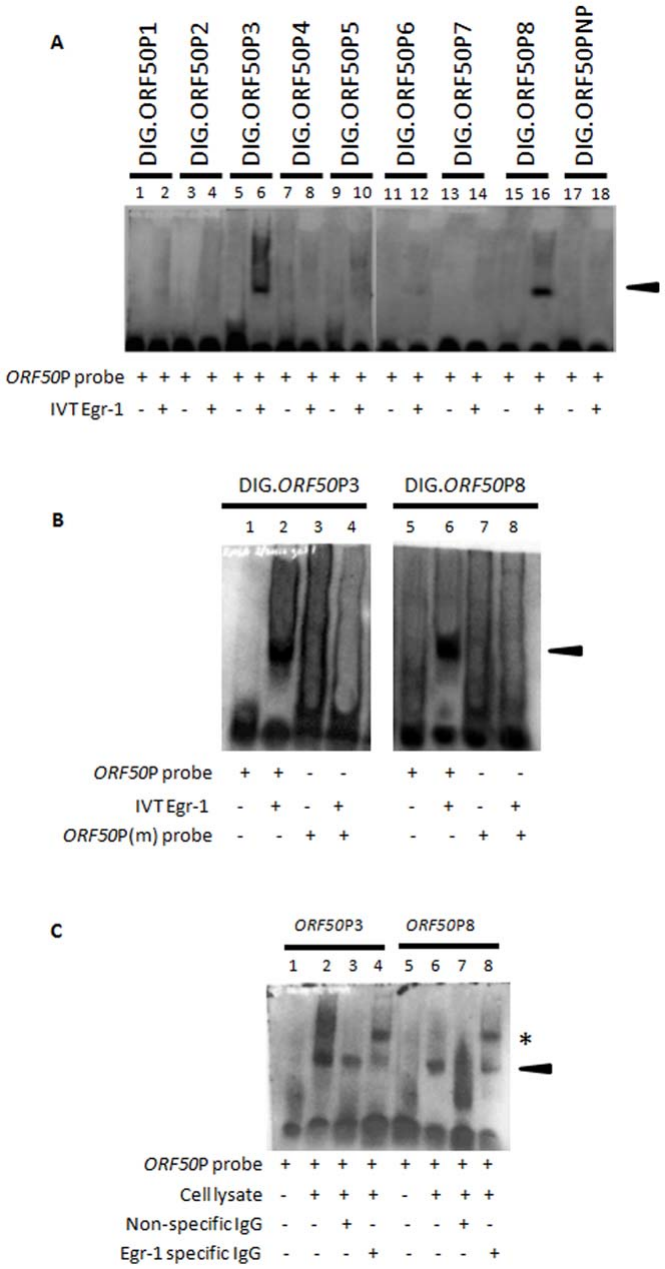
Egr-1 binds two different targets on the KSHV *ORF50*P. (**A**) IVT-synthesized Egr-1 binds to *ORF50*P probes. EMSA studies were performed using IVT-synthesized Egr-1 products and DIG-labeled *ORF50*P probes (see Table 1). (**B**) Mutations in the putative Egr-1 binding domain inhibit Egr-1 binding. EMSA experiments were performed using wt *ORF50*P3 and *ORF50*P8 probes as well as corresponding probes displaying mutations in the suspected Egr-1 binding domain (*ORF50*P3m and *ORF50*P8m). (**C**) Nuclear lysates from KSHV-infected cells formed a complex with *ORF50*P probes. BCBL-1 cells were synchronized in S phase of cell cycle according to earlier protocols [28], treated with 20 ng/ml TPA for 8 h, and lysed. Nuclear extracts containing Egr-1 proteins were used to perform EMSA studies using *ORF50*P3 and *ORF50*P8 probes. Specific Egr-1 binding was confirmed by performing supershifts using specific antibodies to Egr-1 (*lanes 4 and 8*) or nonspecific IgGs (*lanes 3 and 7*). The arrowhead indicates protein/DNA complex formation. Specific antibody/protein/DNA supershifts are denoted by the asterisk.

Egr-1 is said to bind a GC-rich DNA template (such as GCGC(G/T)GGGCG, GCGGGGGCG, and CGCCCATGC) on the promoter and initiate gene transcription [15], [16]. Eight possible GC-rich Egr-1 binding sequences have been identified by us in the promoter region of KSHV *ORF50*. In order to determine the sites where Egr-1 bound *ORF50*P, EMSA experiments were performed using 8 different DIG-labeled probes, referring to identified locations on *ORF50*P, (Table 1) and Egr-1 *in vitro* transcribed and translated (IVT) proteins. IVT of *egr-1*/pcDNA3.1(+) construct yielded a protein of roughly 78 kda (data not shown) [6]. Of all the probes tested, IVT-synthesized Egr-1 proteins were able to bind and form separate protein:DNA complexes with the *ORF50*P3 and *ORF50*P8 probes, respectively; displaying distinct band shifts when compared to controls using the probes alone (Fig. 1A; *lanes 6 and 16*). It is important to note that the sequence for *ORF50*P3 is the same for the probe used in earlier study [6]. Band shifts were not observed when *ORF50*P probes were incubated with IVT-synthesized KSHV glycoprotein L (gL) (*data not shown*). Additionally, experiments using the *ORF50*PNP probe (does not contain the GC-rich binding domain) did not form a complex with Egr-1 protein, thus confirming the specificity of Egr-1 binding (Fig. 1A, lane 18). Finally, competition experiments using unlabeled *ORF50*P probes reduced band shifts by preventing Egr-1 binding to DIG-labeled probes (*data not shown*).

**Table 1:** KSHV *ORF50*P Region 3087 bp (68608–71694 as per NC_009333.1).

Probe	Sequence	Location on ORF50
*ORF50*P1	atggccttgcgcccccacaggagaa	^−^2766 - ^−^2742
*ORF50*P2	tgagccggccctccccttctccacc	^−^2665 - ^−^2641
*ORF50*P3	tttgacctgcgtgcgctctccggct	^−^2173 - ^−^2149
*ORF50*P3m	tttgacctatatacgctctccggct	^−^2173 - ^−^2149
*ORF50*P4	tatgccggggtgcgcgggggtcccg	^−^1320 - ^−^1296
*ORF50*P5	tttcctggtggggcgcggcagctga	^−^994 - ^−^970
*ORF50*P6	ctctgcccatgggcgggtgggtgac	^−^952 - ^−^928
*ORF50*P7	tcattaagccccgcccagaaaccag	^−^119 - ^−^95
*ORF50*P8	aaccagtagctgggtggcaatgaca	^−^100 - ^−^76
*ORF50*P8m	aaccagtagctatatagcaatgaca	^−^100 - ^−^76
*ORF50*NP	caaatagtcgttggctaggttaaag	^−^2911 - ^−^2887

A second set of EMSA studies were conducted using *ORF50*P probes carrying mutations (*ORF50*P3m and *ORF50*P8m; Table 1) in the putative Egr-1 binding region to further confirm the binding ability of Egr-1 proteins. Briefly, *ORF50*P3 and *ORF50*P8 were mutated to carry the 5 bp ATATA sequence in the GC-rich binding domain and then incubated in binding buffer alone or with IVT-synthesized Egr-1. Samples consisting of wildtype (wt) or mutated probes alone did not display a shift in the respective *ORF50*P3 and *ORF50*P8 probes (Fig. 1B; *lanes 1, 3, 5. and 7*). Following incubation of wt probes with Egr-1, complexes were formed producing separate band shifts (Fig. 1B, *lane 2 and 6*). Interestingly, Egr-1 did not bind *ORF50*P3m or *ORF50*P8m probes that displayed mutations in the Egr-1 binding domain, thus confirming the necessity for the consensus GC-rich binding domain mediating Egr-1/*ORF50*P interactions (Fig. 1B, *lanes 4 and 8*).

Finally, gel shift assays were performed using the nuclear extract from KSHV-infected cells to verify the ability of Egr-1 to bind *ORF50*P3 and *ORF50*P8. As expected, there was no hindrance in the migration of *ORF50*P probes without the addition of cell lysate (Fig. 1C; *lanes 1 and 5*). However, the presence of the lysate in the samples resulted in the formation of protein:DNA complexes indicated by a band shift (Fig. 1C, *lanes 2 and 6*). Egr-1 binding to *ORF50*P was confirmed by incubating lysates with specific Abs and performing a supershift. Samples that were incubated with nonspecific IgG Abs displayed band shifts that were similar to samples containing only Egr-1 and the respective probes (Fig. 1C, *lanes 3 and 7*). Alternatively, a supershift occurred exhibiting a discrete band when nuclear lysates were pre-treated with Egr-1 specific Abs (Fig. 1C, *lanes 4 and 8*). Taken together, these experiments provide support for the ability of Egr-1 to specifically bind to two separate locations on KSHV *ORF50*P.

### Egr-1 specifically targets KSHV ORF50P in PEL cells

**Figure 2:**
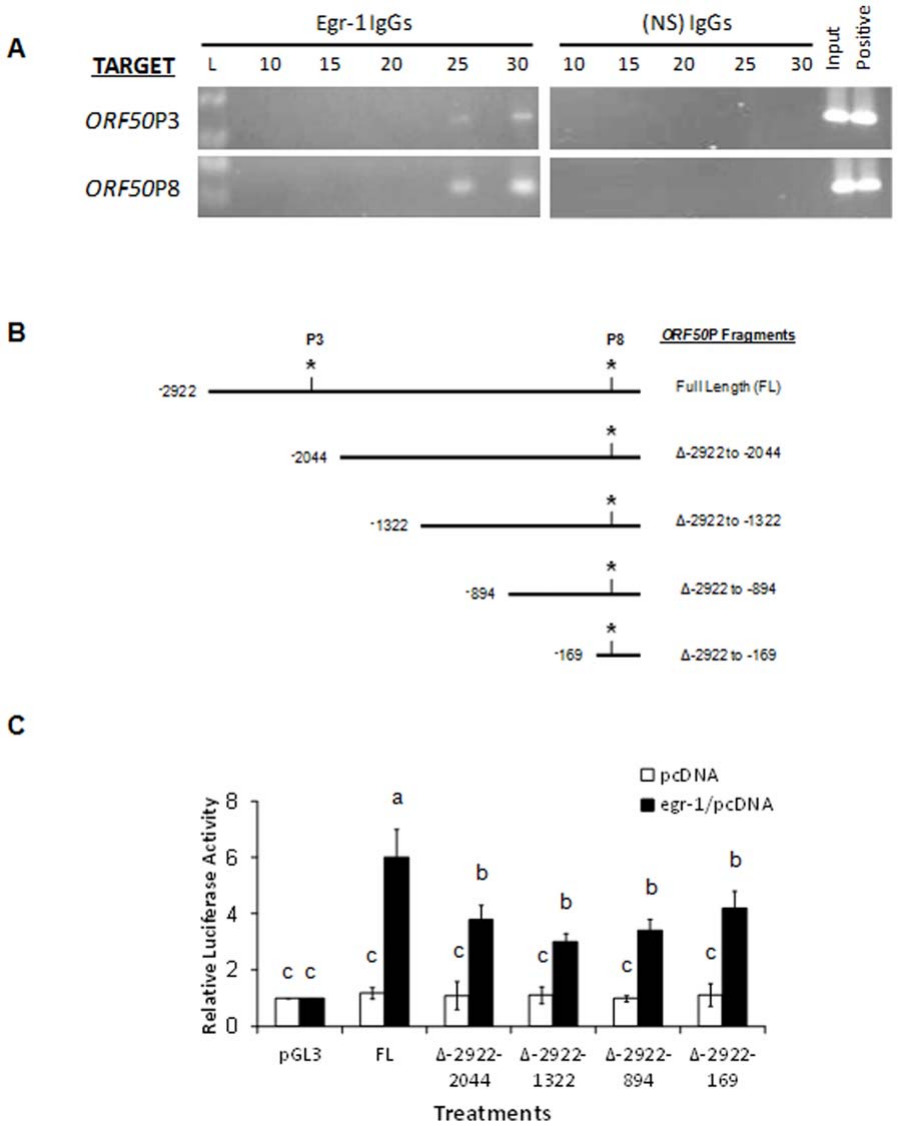
Egr-1 targets KSHV *ORF50 in vivo*. (**A**) BCBL-1 cells were synchronized in S phase [28] and treated with 20 ng/ml of TPA for 8 h. ChIP assays were performed using 2 µg of specific antibodies to Egr-1 or nonspecific IgGs. Primers specifically targeting *ORF50*P3 or *ORF50*P8 (see Table 1) were used to perform semi-quantitative PCR experiments on 1% of total DNA (input) and IP samples. Respective cDNA at 10, 15, 20, 25, and 30 cycles were removed and resolved on a 2% agarose gel. IP of BCBL-1 DNA using specific antibodies to histone H3 was used as positive controls. (**B**) A schematic representation of *ORF50P* used to make the deletions of the luciferase reporter constructs. The nucleotide locations correspond to the old KSHV genome sequence NC_003409 which has since been updated to NC_009333.1. Asterisks refer to the *ORF50P3* and *ORF50P8* locations, respectively. (**C**) Overexpression of Egr-1 activates *ORF50P* via interacting with *ORF50P3* and *ORF50P8* domains. HEK293 cells were co-transfected with a combination of three vectors, one from the following groups: (i) pcDNA3.1(+) or *egr-1*/pcDNA3.1(+), (ii) the control vector, pRL-TK, and (iii) empty pGL3 vectors or pGL3 vectors encoding FL *ORF50P* or one of several deletions (Δ-2922 to -2044; Δ-2922 to -1322; Δ-2922 to -894; and Δ-2922 to -169). After 48 h post-transfection, the cells were lysed, and relative luciferase activity was monitored. Firefly luciferase was normalized to the corresponding *Renilla* luciferase activity. The luciferase activation of pGL3 by *egr-1*/pcDNA3.1(+) was represented as 1-fold. Each point denotes the average ± SD of three experiments. Columns with different *alphabets* are statistically significant (P<0.05) by least significant difference (LSD).

A semi-quantitative chromatin immunoprecipitation (ChIP) assay was performed to analyze Egr-1 binding to *ORF50*P in a chromatin context (*in vivo*) using specific antibodies. TPA-induced KSHV-infected cells were used to assess the binding ability of Egr-1 to *ORF50*P via ChIP assays. The presence of specific *ORF50*P in the IP samples was analyzed by semiquantitative PCR using specific primers covering the regions of *ORF50*P3 or *ORF50*P8. As expected, when Egr-1 was expressed in BCBL-1 cells it was recruited to the promoter of KSHV *ORF50* and specifically targeted both *ORF50*P3 and *ORF50*P8 (Fig. 2A, *cycle 30*). Recruitment of Egr-1 to the nonspecific *ORF50*NP region was not detectable in our experiments (data not shown). For negative controls, samples were IP with nonspecific (NS) IgG Abs and recruitment of Egr-1 to *ORF50*P was not observed (Fig. 2A, *cycle 30 on control gels*). However, positive controls using specific Abs to histone proteins recovered *ORF50*P targets (Fig. 2A). These results help us confirm that Egr-1 binds to two separate domains on *ORF50*P, *in vivo*.

To establish a critical role for these interactions between Egr-1 and *ORF50*P, luciferase reporter constructs were used to investigate the necessity of *ORF50*P3 and *ORF50*P8 during Egr-1-mediated activation of the *ORF50*P. Empty vector (pGL3) or vectors encoding a deletion series of *ORF50*P (Fig. 2B) along with the downsteam luciferase gene were transiently transfected into target cells in conjunction with empty vector or *egr-1*/pcDNA3.1(+). Cells transfected with pcDNA3.1(+) did not induce significant luciferase activity (Fig. 2C). However, following incubation of cells transfected with *egr-1*/pcDNA3.1(+), we noticed the luciferase activity to be significantly greater in cells that were also transfected with constructs encoding the full length (FL) *ORF50*P compared to cells transfected with pGL3 (Fig. 2C), Furthermore, we observed a decrease in relative luciferase activity following deletion of the fragment containing the *ORF50*P3 domain (Δ-2922 to -2044; Δ-2922 to -1322; Δ-2922 to -894; and Δ-2922 to -169) when compared to the construct encoding full length *ORF50*P (Fig. 2C). Although the absence of *ORF50*P3 contributed to a decrease in luciferase activity, this activity was never completely abolished (Fig. 2C) suggesting the need for an intact *ORF50*P3 and *ORF50*P8 for an optimal Egr-1-induced transcription of *ORF50*. Taken together, these results suggest a role for Egr-1 to specifically bind and activate *ORF50*P to trigger a lytic infection in KSHV-infected cells.

### Cellular Egr-1 and virus-encoded KSHV RTA follow a similar expression pattern during *de novo* KSHV infection

**Figure 3:**
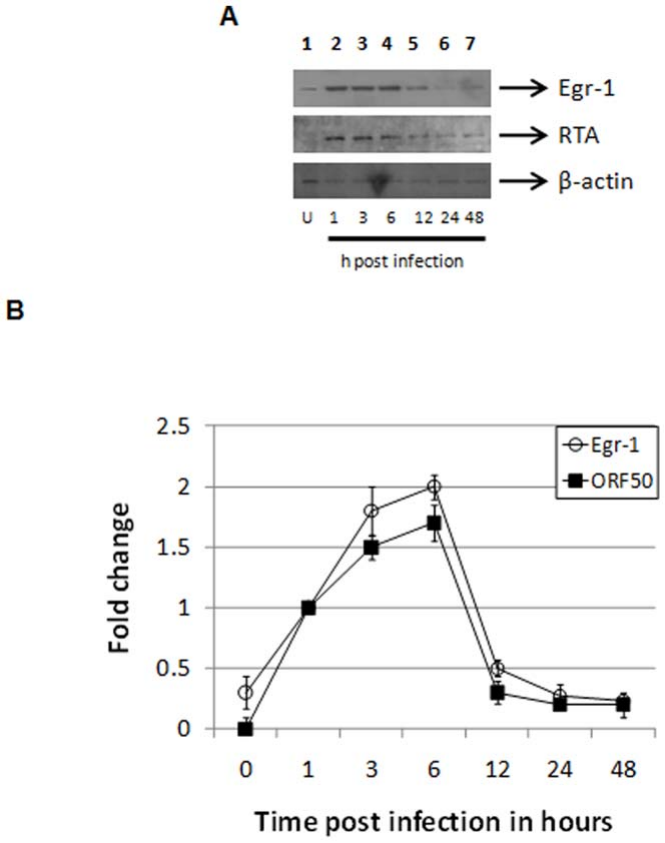
KSHV RTA and Egr-1 follow a similar expression pattern during *de novo* KSHV infection. HEK293 cells were infected with KSHV at 5 MOI, incubated at 37°C at different time points up to 48 hPI, and lysed. (**A**) Expression of KSHV RTA and Egr-1 were elevated up to 6 hPI. SDS-PAGE was performed using uninfected (*lane 1*) or KSHV-infected cell lysates (*lanes 2–7*). The samples were run on a 10% gel and transferred to a PVDF membrane. Western blotting was performed using specific Egr-1 or KSHV RTA Abs. Abs targeting β-actin were used as internal controls. (**B**) Analysis of *ORF50* and *egr-1* transcription. KSHV-infected HEK293 cells were lysed and RNA was extracted. Next, cDNA was synthesized and subjected to quantitative real-time PCR analysis. Baseline expression of genes at 1 hPI was considered as 1-fold for comparisons. Each point denotes the average±S.D. (*error bars*) of three experiments.

Several different viruses are known to activate Egr-1 expression upon infection [17], [18], [19], [20]. Since BCBL-1 cells already carry KSHV DNA, KSHV-infected HEK293 cells were used to evaluate the expression pattern of Egr-1 and KSHV RTA during early stages of *de novo* infection. Expression of Egr-1 and RTA proteins were significantly elevated by 1 hour post infection (hPI) and continued to maintain increased expression until roughly 6–8 hPI (Fig. 3A, *lanes 2–4*). In contrast, a considerable decrease in the expression of these proteins was observed from 12–48 hPI (Fig. 3A, *lanes 5–7*). A significant difference in expression of total β-actin was not observed (Fig. 3A) during the course of KSHV infection demonstrating the specificity of the results on Egr-1 and RTA expression.

To further support our findings, mRNA extracted from KSHV-infected HEK293 cells were subjected to quantitative real-time PCR (qRT-PCR) analysis in order to evaluate *egr-1* and *ORF50* transcriptional activity. Uninfected cells (0 hPI) did not show detectable *ORF50* expression (Fig. 3B). On the other hand, a low baseline level of *egr-1* expression was observed in the uninfected samples (Fig. 3B). With the onset of a primary infection, expression levels of both *egr-1* and *ORF50* increased up to 6hPI (Fig. 3B). These elevated levels of *egr-1* and *ORF50* decreased substantially by 12–24 hPI (Fig. 3B). No significant changes in the expression of the internal control gene encoding *M6PR* was observed indicating specificity of the results (data not shown). Taken together, the expression profiles of Egr-1 and RTA seem to follow an identical pattern during primary infection of cells.

### Elevated Egr-1 expression activates lytic genes in KSHV-infected cells

**Figure 4:**
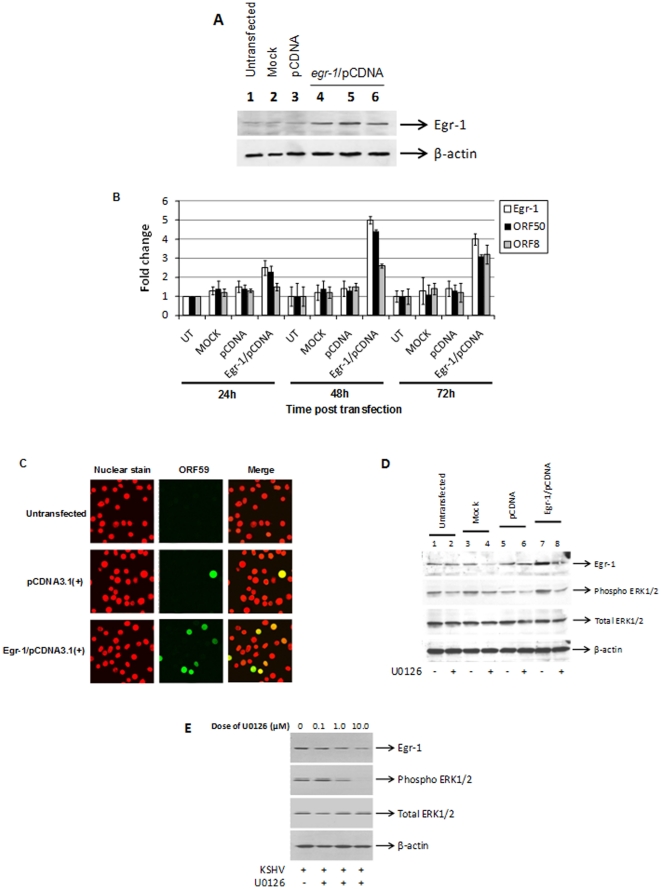
Elevated Egr-1 induces KSHV lytic gene expression. (**A**) Elevated Egr-1 was observed in cells transfected with egr-1/pCDNA3.1(+). BCBL-1 cells were untransfected, mock transfected, transfected with pcDNA3.1 or *egr-1*/pcDNA3.1. The cells to be used as controls (*lanes 1–3*) were incubated at 37°C for 48 h while the cells transfected with *egr-1*/pcDNA3.1 were incubated for 24, 48, and 72 h, respectively. At the end of incubation, the cells were lysed and the lysates were used to perform Western blotting. (**B**) Effect of elevated Egr-1 on KSHV *ORF50* and *ORF8* expression. BCBL-1 cells were untransfected or transfected as described above. RNA was extracted, cDNA was synthesized, and the expression of cellular *egr-1*; and KSHV encoded *ORF50* and *ORF8* were analyzed by qRT-PCR. Baseline expression of genes was considered as 1-fold for comparisons. Each point denotes the average±S.D. (*error bars*) of three experiments. (**C**) Expression of lytic proteins in BCBL-1 cells transfected with Egr-1. KSHV-infected cells were untransfected, transfected with pcDNA3.1, or *egr-1*/pcDNA3.1 for 48 h. The stained cells examined using a confocal microscope (magnification ×62). The average number of fluorescent cells were counted over five random fields and used for comparisons. (**D**) Enhanced egr-1 activates MAPK signaling in BCBL-1 cells. KSHV-infected cells were untransfected, mock-transfected, transfected with pcDNA3.1(+), or *egr-1*/pcDNA3.1(+) for 48 h. Each group of cells was left untreated or they were treated with 10 µM of U0126 1 h prior to transfection and remained throughout the incubation period. Cell lysates were resolved on a 10% SDS-PAGE, transferred to a PVDF membrane, and Western blotting was performed using specific antibodies. (**E**) U0126 inhibits phosho-ERK1/2 and Egr-1. Briefly, BCBL-1 cells were treated with different concentrations of U0126. Following 24 h incubation, the cells were lysed and the lysates were used to perform Western blotting as per earlier protocols using specific antibodies.

BCBL-1 cells have turned out to be a blessing in disguise for this study as they harbor KSHV DNA in a predominantly latent state [21]. BCBL-1 cells were transiently transfected using *egr-1*/pCDNA3.1(+) for 24, 48, and 72 h (Fig. 4A; *lanes 4, 5, 6*). The highest expression of Egr-1 was observed by 48 h post transfection; followed by a reduction in the next 24 h. Target cells that were untransfected (Fig. 4A; *lane 1*), mock transfected (Fig. 4A; *lane 2*), and transfected with empty vectors (Fig. 4A; *lane 3*) did not display an observable difference in Egr-1 expression. Next, qRT-PCR studies were performed to identify changes in *egr-1* and virus-encoded *ORF50* gene expression. We did not observe any noticeable alterations in *egr-1* and *ORF50* transcription in target cells that were untransfected (UT), mock transfected, or transfected with empty vectors (Fig. 4B). As expected, levels of *egr-1* were significantly increased in cells transfected with *egr-1*/pCDNA3.1(+) over controls (Fig. 4B). Furthermore, elevated *egr-1* expression coincided with an increase in KSHV *ORF50* transcription (Fig. 4B). Peak expression for both genes was observed by 48 h post transfection (Fig. 4B). Incidentally, we also observed an elevated expression of *ORF8* (Fig. 4B), encoding the late structural virus protein termed as gB, in response to an enhanced *egr-1* and *ORF50* expression. All these changes implicate Egr-1 expression to significantly induce virus reactivation.

Immunofluorescence assay (IFA) was conducted to determine a possible role for elevated Egr-1 on the expression of KSHV-encoded lytic proteins in the above transfected cells (Fig. 4A, B). KSHV-encoded ORF59, a processivity factor for KSHV DNA polymerase, is expressed in the nucleus of infected cells during early stages of virus reactivation [22]. Target cells transfected with empty vectors showed low levels of ORF59 expression (Fig. 4C). However, transfection cells with *egr-1*/pcDNA3.1(+) augmented the level of ORF59 protein expression in KSHV-infected cells (Fig. 4C); clearly implicating a critical role for Egr-1 in inducing KSHV reactivation.

Finally, we analyzed MAPK signaling in the above cells relative to Egr-1 expression levels. Our results suggest that cells transfected with *egr-1*/pCDNA3.1(+) displayed elevated levels of Egr-1 (Fig. 4D, *lane 7*). Transfection of cells with pCDNA3.1(+) (Fig. 4D, *lane 5*) or mock transfection (Fig. 4D, *lane 3*) of cells did not significantly alter the expression levels of Egr-1 and phosphorylation state of ERK1/2 compared to untransfected cells (Fig. 4D, *lane 1*). Interestingly, treatment of cells with a known inhibitor of MEK1/2 (10 µM of U0126) significantly lowered both the expression levels of Egr-1 and ERK1/2 activity (Fig. 4D, *lane 8*). We observed U0126 to dose dependently inhibit ERK1/2 activity and Egr-1 levels in the above cells confirming the specificity of U0126 in targeting MAPK>Egr-1 signaling. The inhibition of ERK1/2 activity and Egr-1 levels was greatest following treatment of infected cells with 10 µM of U0126 (Fig. 4E, *lane 4*). These results suggest Egr-1 to be downstream of the MAPK signaling cascade.

### Resveratrol inhibits Egr-1 and ORF50 during early and late stages of infection

**Figure 5:**
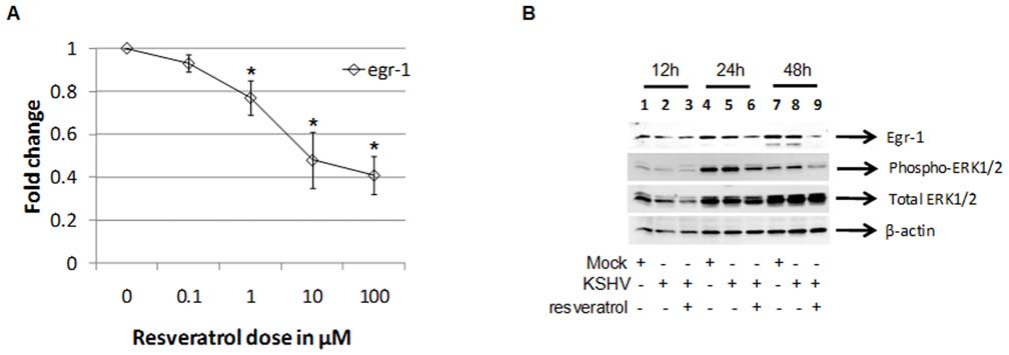
Resveratrol inhibits Egr-1 and KSHV *ORF50* expression during early stages of infection. (**A**) Resveratrol inhibited *egr-1* expression by as early as 6 hPI in HEK293 cells. HEK293 cells were infected with 5 MOI of KSHV for 2 h at 37°C. After infection, the cells were washed and cultured in growth medium in the presence or absence of different concentrations of resveratrol for 4 h. At the end of incubation, the cells were lysed, RNA was extracted, cDNA was synthesized, and *egr-1* levels were analyzed by qRT-PCR. Each point denotes the average±S.D. (*error bars*) of three experiments. (**B**) Resveratrol inhibits MAPK activity during *de novo* KSHV infection. HEK293 cells were mock-infected or infected with 5 MOI of KSHV for 2 h at 37°C. After infection, the cells were washed and cultured in growth medium in the presence or absence of 100 µM of resveratrol for 48 h. The cells were lysed using gold lysis buffer (GLB) and the lysates were resolved on a 10% SDS-PAGE, transferred to a PVDF membrane, and Western blotting was performed using specific antibodies.

Due to the vital role of MAPK signaling on Egr-1 expression and KSHV reactivation, the effect of resveratrol on KSHV replication was analyzed. We chose to use resveratrol because: (i) it is a naturally occurring product; and (ii) it is a known regulator of MAPK signaling and Egr-1 expression [14]. Furthermore, resveratrol inhibits ERK1/2 activity in virus-infected cells [23]. It is important to understand that even though KSHV-encoded *ORF50* is a gene crucial for reactivation, it is also expressed during early stages of KSHV infection and may play a role in the establishment of virus latency [6], [24]. Therefore, it was necessary to determine the expression pattern of cellular *egr-1* and virus-encoded *ORF50* during both early stages of infection as well as during virus reactivation (late stages). In this study, effect of resveratrol on early stages of infection was analyzed in HEK293 cells while its effect on late stages (reactivation using TPA) was analyzed predominantly in BCBl-1 cells, for convenience. In this study, resveratrol was able to inhibit *egr-1* expression in a dose dependent manner during early stages of *de novo* KSHV infection of HEK293 cells (Fig. 5A). The doses tested in this study were confirmed by trypan blue test to be non-toxic to cells (data not shown). The resveratrol doses used by us are also those that have been published previously [25], [26], [27]. Resveratrol (100 µM) was able to suppress the expression of phospho-ERK1/2 and Egr-1 proteins during *de novo* infection of cells (Fig. 5B, *lanes 3, 6, and 9*). Additionally, resveratrol was able to inhibit the expression Egr-1 and phophorylated ERK1/2 in mock-infected cells (Figure S1, *lanes 2, 4, and 6*). On the other hand, it was not able to significantly alter the expression of endogenous ERK1/2 and actin controls (Fig. 5B, *lanes 3, 6, and 9*; Figure S1, *lanes 2, 4, and 6*). DMSO, the vehicle for resveratrol, did not significantly alter the phosphorylation of ERK1/2 and the expression of Egr-1 (data not shown).

**Figure 6:**
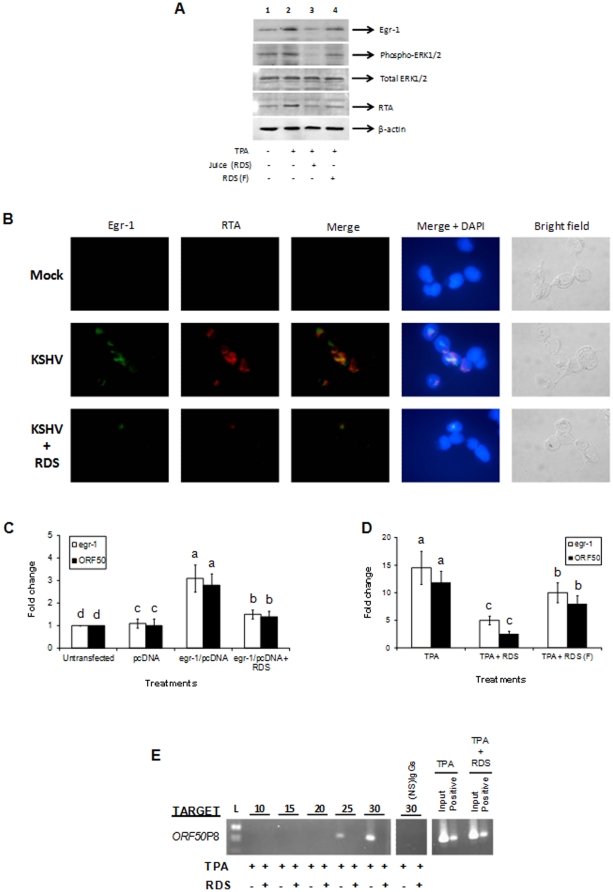
RDS reduces the Egr-1/*ORF50* association *in vivo*. (**A**) RDS lowers Egr-1 and KSHV RTA expression. KSHV-infected BCBL-1 cells were synchronized in S phase and untreated or treated using 20 ng/ml of TPA for 2 h. Each group of cells was left untreated or was further treated using filtered or unfiltered RDS containing 100 µM of resveratrol. The cells were incubated at 37°C for 6 h and lysed. The lysates were resolved on a 10% SDS-PAGE, transferred to a PVDF membrane, and Western blotting was performed using specific antibodies. (**B**) RDS reduces the number of KSHV-infected cells undergoing reactivation in HEK293 cells. Mock-infected, KSHV-infected, and KSHV-infected cells in the presence of RDS containing 100 µM resveratrol were stained using monoclonal mouse anti-Egr-1 IgGs and rabbit peptide antibodies targeting KSHV RTA and examined under a fluorescent microscope (magnification ×100). (**C**) Overexpression of Egr-1 is unable to overcome RDS-mediated inhibition of virus reactivation. BCBL-1 cells were transiently transfected using pcDNA3.1(+) or *egr-1*/pcDNA3.1(+) and subsequently treated with unfiltered RDS containing 100 µM of Resveratrol for 6 h. RNA was extracted, cDNA was synthesized, and *egr-1* and KSHV *ORF50* were analyzed by qRT-PCR. Each point denotes the average±S.D. (*error bars*) of three experiments. (**D**) RDS lowers egr-1 and KSHV *ORF50* transcriptional activity. BCBL-1 cells were synchronized in S phase, treated with 20 ng/ml of TPA, and treated using filtered or unfiltered RDS containing 100 µM of resveratrol as before. RNA was extracted, cDNA was synthesized, and *egr-1* and KSHV *ORF50* were analyzed by qRT-PCR. Each point denotes the average±S.D. (*error bars*) of three experiments. Columns with different *alphabets* are statistically significant (P<0.05) by LSD. (**E**) RDS inhibits the ability of Egr-1 to bind KSHV *ORF50 in vivo*. BCBL-1 cells were synchronized and treated with TPA as before. The cells were further treated using unfiltered RDS containing 100 µM of resveratrol and incubated for 6 h. ChIP assays were performed using 2 µg of specific Egr-1 Abs. Semi-quantitative PCR experiments were performed using samples from 1% input DNA and IP samples in order to determine the expression of *ORF50*P8. Respective cDNA at 10, 15, 20, 25, and 30 cycles were resolved on a 2% agarose gel. Specific antibodies to histone H3 and nonspecific IgGs were used to IP sample chromatin and served as positive and negative controls, respectively.

In order to present more physiologically relevant studies, an over-the-counter resveratrol dietary supplement (RDS) was used to treat KSHV-infected BCBL-1 cells under TPA-induced conditions. RDS containing 100 µM resveratrol did not significantly induce cell death as monitored by the lactate dehydrogenase assay (data not shown). These results were confirmed by the conventional trypan blue test. More than 95% of the target cells were found to be viable when the target cells were treated with RDS (data not shown). As shown in earlier reports [12], TPA treatment augments phospho-ERK1/2 expression (Fig. 6A, *lane 2*). The effect of TPA also resulted in an increase in Egr-1 and KSHV RTA expression when compared to uninduced cells (Fig. 6A, *lane 2*). Unfiltered RDS successfully inhibited phospho-ERK1/2, Egr-1, and RTA expression in TPA-induced KSHV-infected cells (Fig. 6A, *lane 3*). A slightly lesser inhibitory effect was observed in cells that were treated with RDS that had particulates removed using a 0.2 µm filter (Fig. 6A, *lane 4*). We did not discover either form of RDS to have noticeable effect on endogenous ERK1/2 and actin controls (Fig. 6A, *lanes 3 and 4*). The data from Western blotting (Fig. 6A) was further confirmed in HEK293 cells by IFA (Fig. 6B). To authenticate the results from monitoring the effect of RDS on protein levels of Egr-1 and RTA, we analyzed the effect of RDS on (i) uninduced cells transfected with vectors encoding *egr-1*, and (ii) TPA-induced KSHV reactivation in BCBL-1 cells by performing qRT-PCR. Our results clearly demonstrate the ability of RDS to lower the expression of both *egr-1* and *ORF50* under both circumstances (Fig. 6C, D). Finally, ChIP assay was performed to discern the specificity of RDS on virus reactivation using primers specific to *ORF50*P8 region. Under TPA-induced conditions, Egr-1 specifically targeted *ORF50*P8 (Fig. 6E. *cycles 25 and 30*). However, under RDS treated conditions the binding of Egr-1 to *ORF50*P8 was significantly decreased (Fig. 6E, *cycles 25 and 30*). For negative controls, samples were IP with (NS) IgGs and recruitment of Egr-1 to *ORF50*P was not observed (Fig. 6E). However, positive controls using specific Abs to histone proteins recovered *ORF50*P targets (Fig. 6E). These results suggest resveratrol in its chemical form and RDS may lower Egr-1 expression to inhibit KSHV reactivation from latency.

## Discussion

Egr-1 regulates expression of several viral genes and plays a crucial role in the replication of different viruses Our results from employing the EMSA and ChIP assay (Fig. 2A) demonstrate that Egr-1 may bind *ORF50*P via at least two different GC-rich binding domains: at positions between ^−^100 – ^−^76 bp (*ORF50*P8) and ^−^2173 – ^−^2149 bp (*ORF50*P3). The results from employing the ChIP assay (Fig. 2A) demonstrate that Egr-1 may bind *ORF50*P with a greater affinity at positions between ^−^100 – ^−^76 bp (*ORF50*P8) compared to ^−^2173 – ^−^2149 bp (*ORF50*P3). However, we did not observe any such differences in the binding affinity of Egr-1 to *ORF50*P3 and *ORF50*P8 by EMSA using IVT Egr-1 (Fig. 1B, C). All the more, our data supports the need for Egr-1 to bind both *ORF50*P3 and *ORF50*P8 for an optimal transcriptional activity in luciferase reporter assays (Fig. 2C). This difference observed in Egr-1 binding to both these domains could be due to one or both the reasons: (i) IVT synthesized Egr-1 was used in EMSA experiments; and (ii) the design of ChIP assay conducted in this study was not to decipher the relative binding affinity of Egr-1 to these domains; instead was performed to just confirm if Egr-1 bound these domains, *in vivo*.

Although we previously noticed that the e*gr-1* and KSHV-encoded *ORF50* followed a similar expression pattern, the experiments were conducted in TPA-induced cells to evaluate their expression during the reactivation process [6]. The present study discovered that the transcriptional activity of *egr-1* and *ORF50* and their subsequent translation is comparable and followed a similar pattern during *de novo* KSHV infection (Fig. 3). However, enhanced cellular Egr-1 and viral RTA expression during early stages of primary infection (Fig. 3) is not sufficient to trigger a lytic infection [6]. These results suggest the following: (i) the role of Egr-1>RTA signaling in initiating a lytic cycle of infection during the course of initial infection of cells is limited; and (ii) there is a missing element in the Egr-1>RTA driven cellular events critical for inducing a productive replication.

In this study, transfection of cells with *egr-1*/pCDNA3.1(+) resulted in a significant increase in virus-encoded *ORF50* transcription followed by the expression of early-lytic ORF59 protein and late-lytic gene (*ORF8*) encoding gB (Fig. 4B, C); all of which are indicators of an active lytic replication of KSHV [12], [28], [29]. MAPK signaling was observed to regulate Egr-1 expression in cells transfected with egr-1/pCDNA3.1(+) (Fig. 4D). Interestingly, treatment of KSHV-infected cells with TPA induces a lytic replication via MAPK signaling [12], [13]. In addition, Egr-1 is a downstream target of Raf/MEK/ERK signaling (Fig. 4D) [6], [30]. It is unclear at this time if the effect of *egr-1*/pcDNA3.1 over-expression resembles the milieu supporting a lytic infection *in vivo*. It is important to remember that KSHV reactivation can be regulated by other cellular factors including STAT6, NFκB, and XBP-1 [31], [32], [33]. Thus, activation of a lytic infection may require unique cellular factors under specific conditions or a combination of factors. Further investigation is required to unravel the environment(s) supporting virus reactivation under physiologically relevant conditions; especially in terms of the different transcription factors.

In order to support these findings, more physiological relevant studies were performed by analyzing the effect of resveratrol on KSHV-infected cells. Resveratrol, or trans-3,5,4′-trihydroxystilbene, is a phytoalexin that is produced in various plants such as grapes, berries, and peanuts in response to attacks by pathogens [14]. Several reports provide evidence for resveratrol to exhibit antiviral effects [27], [34], [35]. On the other hand, resveratrol has also been shown to induce virus replication [36], [37]. We have demonstrated that resveratrol, in its chemical form, inhibits Egr-1 and phospho-ERK1/2 in KSHV-infected cells (Fig. 5B). RDS significantly inhibited KSHV reactivation in uninduced and TPA-induced cells (Fig. 6A, B, C, D). While performing these experiments we noticed that unfiltered RDS was able to inhibit KSHV reactivation to a greater extent when compared to cells treated with RDS that was passed through a 0.2 µm filter. However, both treatments were able to significantly inhibit gene products associated with KSHV reactivation (Fig. 6A, D). These differences are likely due to the presence of unknown factors in unfiltered RDS that may act in combination with resveratrol. Incidentally, the decrease in the expression of RTA by RDS coincided with a sharp decline in the ability of Egr-1 to bind *ORF50*P as shown by the semi-quantitative ChIP assay (Fig. 6E). Taken together, this is the first report to describe the role of physiologically relevant RDS on KSHV infection.

Several cellular pathways are regulated by resveratrol including apoptotic, NFκB, and all forms of MAPK signaling [14], [38]. We found resveratrol to inhibit expression of Egr-1 and phosphorylation of ERK1/2 resulting in suppression of KSHV reactivation (Fig. 5 and 6). Very recent studies have established the fact that resveratrol significantly lowers phosphorylation of ERK1/2 (directly upstream of Egr-1) in target cells, *in vivo* and *in vitro* [25], [26], [39], [40]. At this point in time, we are certain about the ability of RDS to block TPA-induced virus reactivation. However, further research is required to confirm if this ability of RDS to promote viral latency is by its direct inhibitory effect on the expression Egr-1 or the upstream MAPK signaling component(s), namely ERK1/2 activity.

KSHV reactivation from latency is a complicated process which is regulated by an intricate relationship between viral and cellular factors. The method in which resveratrol may regulate KSHV reactivation has yet to be fully understood. However, we propose that resveratrol may inhibit KSHV reactivation by altering the interactions between cellular Egr-1 and viral *ORF50*P in a Raf>MEK>ERK-dependent manner. All three MAPKs (ERK, p38, and JNK) have been shown to positively regulate Egr-1 expression [41]. However, the role for active p38 MAPK is not fully understood as it has been shown to reduce Egr-1 expression in B-lymphocytes unlike ERK and JNK [42]. Further studies are required to better understand the involvement of different MAPKs on Egr-1 expression during KSHV infection. The findings presented in this study open a Pandora's Box of questions pertaining to treating/managing a variety of viral infections using RDS. Future studies are aimed at appreciating the cellular milieu critical for the effectiveness of the MAPK associated signaling in inducing virus reactivation. These findings may provide for more useful applications to combat a variety of viral lytic infections.

## Materials and Methods

### Cells

HEK293 cells and BCBL-1 cells were cultured in DMEM and RPMI (Invitrogen, Carlsbad, CA), respectively, as per earlier studies [6], [43].

### Antibodies

Rabbit antibodies to gB [44], RTA [6]; and mouse antibodies to ORF59 (Bryan et al., 2006) were used in this study. Rabbit antibodies to phospho-ERK1/2, total ERK1/2, actin, and Egr-1 (15F7; monoclonal antibodies) purchased from Cell Signaling Technology, Beverly, MA were used in this study. Mouse (S-25) and rabbit (15F7) antibodies to Egr-1 purchased from Santa Cruz biotechnologies, Inc. (Santa Cruz, CA) were used in Immunofluorescence assay (IFA) and Western blotting experiments, respectively. Additionally, rabbit polyclonal antibodies (588) to Egr-1 was used in gel shift and chromatin immunoprecipitation assays (Santa Cruz Biotechnology, Santa Cruz, CA).

### Vectors

We used *egr-1*/pCDNA3.1(+) and gL/pCDNA3.1(+) vectors in this study. Both these vectors have been described elsewhere [6].

### Reagents

U0126 (Promega, Madison, WI) and Resveratrol (Enzo Life Sciences, Plymouth Meeting, PA), resuspended in DMSO, and stored at −20°C. Finally, 4Resveratrol™ liquid supplement (Genesis Today, Austin, TX) was filtered with a 0.2 µm filter or left unfiltered and used in this study. 4Resveratrol™ dietary supplement (RDS) contains 575 mg of resveratrol per 1 oz of serving. In all of this study, we used RDS at a final concentration of 100 µM.

### *In vitro* transcription and translation (IVT)

IVT of *egr-1*/pCDNA3.1(+) and gL/pCDNA3.1(+) was conducted as per earlier studies [45] using the TNT-coupled rabbit reticulocyte lysate system (Promega).

### Primary infection of HEK293 cells

HEK293 cells were infected as per earlier procedures (25).

### Sorting of cells in different phases of the cell cycle

In this study, we synchronized BCBL-1 cells in S phase of cell cycle as per earlier protocols [46].

### Western blotting

Equal amounts (20 µg) of protein was used in Western blotting experiments as per earlier studies [47].

### qRT-PCR

The qRT-PCR was performed using the synthesized cDNA in a 25 µl reaction volume to analyze the expression of *ORF50*, *egr-1*, and *M6PR* as per earlier protocols [47].

### Transfection of target cells

Target cells were transfected with pCDNA3.1(+), *egr-1*/pCDNA3.1(+), gL/pCDNA3.1(+) using GeneJammer transfection reagent (Stratagene, La Jolla, CA) as per earlier studies [12].

### IFA

Target cells were fixed for 10 min in ice cold acetone and washed thrice in phosphate buffered saline (PBS). These cells were sequentially stained with mouse anti-ORF59 antibodies and goat anti-mouse FITC as per earlier studies [12]. The stained cells were further incubated for 20 min on ice with 5 mM SYTO Red (a nuclear stain; Invitrogen) before being analyzed with a laser-scanning LSM 510 Carl Zeiss confocal microscope. In another set of experiments, acetone fixed cells were incubated with a combination of mouse anti-Egr-1 IgGs and rabbit anti-RTA for 45 min at room temperature (RT), and incubated with a combination of goat anti-mouse FITC and goat anti-rabbit TRITC) for 30 min at RT. Stained cells were washed in PBS, mounted by using an anti-fade reagent containing DAPI (4,6-diamidino-2-phenylindole; Molecular Probes) and examined under a Nikon fluorescent microscope with appropriate filters.

### EMSA

IVT products of Egr-1 or KSHV gL were evaluated by EMSA for DNA binding using several 25 bp digoxygenin (DIG)-labeled probes containing sequences from the *ORF50*P (Table 1) as per earlier studies [6]. For a supershift, the cellular lysate was incubated with rabbit monoclonal antibodies to Egr-1 or nonspecific IgG at 37°C for 30 min prior to the addition of the DIG-labeled probe. All samples were run on a 4% non- denaturing gel for approximately 1.5 h and transferred to a PVDF membrane. The protein:DNA interaction was detected using the CSPD detection system (Roche Applied Science).

### Luciferase Assay

Target cells were transiently co-transfected using appropriate pGL3 and internal control pRL-TK contructs (Promega) and pcDNA3.1(+) vectors (Invitrogen). The total amount of DNA used per sample was approximately 2 µg. Following 48 h post-transfection, cells were harvested and Firefly and *Renilla* luciferase activities were analyzed using the dual luciferase system (Promega). Luciferase activity was monitored using a Turner Systems Luminometer (Sunnyvale, CA) as per earlier protocols [6]. The relative luciferase activity was calculated by normalizing *ORF50*P luciferase activity to control *Renilla* luciferase activity. The results were plotted as a percentage of the activity of the empty pGL3 vector.

### Chromatin Immunoprecipitation Assay

BCBL-1 cells were treated with a final concentration of 1% formaldehyde and crosslinked for 10 min at RT. Crosslinking was stopped by addition of glycine at a final concentration of 0.125 M for 5 min. The cross-linked cells were washed in 1× PBS and counted so that approximately 10^7^ cells were used in each immunoprecipitation (IP) reactions. Nuclei from the cells were purified and lysed to collect chromatin. Chromatin was sheared to approximately 500 bp using a Bioruptor sonicator (Diagenode, Sparta, NJ). Lysates containing the chromatin were pre-cleared using 35 µl of Protein A sepharose beads (Amersham Biosciences) in pre-IP dilution buffer for 30 min at 4°C. The samples were centrifuged to remove beads and the lysate was recovered. After setting aside input controls, primary antibodies were added to the samples and incubated overnight at 4°C. The DNA/protein complexes were IP using protein A beads for 4 h at 4°C and then washed using various ChIP wash buffers. Following elution, proteinase K was added to the complexes and incubated at 65°C overnight in order to reverse the crosslinks. The DNA samples were purified using phenol/chloroform extraction, resuspended in 1× TE buffer, and finally analyzed by polymerase chain reaction (PCR). PCR was performed using platinum pfx polymerase (Invitrogen, Carlsbad, CA) as per standard protocols. PCR amplification of the precipitated DNA was performed using primers that targeted *ORF50*P3, *ORF50*P8, and *ORF50*PNP regions (Table 1): *ORF50*P3 forward, 5′-TTCCCTTTTGACCTGCGTGCG-3′ and reverse, 5′- CGAAGTTTGACGGCCTATACTGTAGG-3′ (178 bp product); *ORF50*P8 forward, 5′-CTACCGGCGACTCATTAAGC-3′, and reverse, 5′-GTGGCTGCCTGGACAGTATT-3′ (126 bp product); *ORF50*PNP forward, 5′-CTAGGGGCGGAAATTTACAA-3′, and reverse, 5′-GGTTCCAGGGCTGTAATCACT-3′ (131 bp product).

## Supporting Information

Figure S1 **Resveratrol inhibits Egr-1 expression in the absence of KSHV infection.** HEK293 cells were mock-infected by incubating with growth medium for 2 h at 37°C. These cells were washed and cultured in growth medium in the presence or absence of 100 µM of resveratrol for 48 h. The cells were lysed using gold lysis buffer (GLB) and the lysates were resolved on a 10% SDS-PAGE, transferred to a PVDF membrane, and Western blotting was performed using specific antibodies.
(TIF)

## References

[1] Chang Y, Cesarman E, Pessin MS, Lee F, Culpepper J (1994). Identification of herpesvirus-like DNA sequences in AIDS-associated Kaposi's sarcoma.. Science.

[2] Ackermann M (2006). Pathogenesis of gammaherpesvirus infections.. Vet Microbiol.

[3] de Sanjose S, Mbisa G, Perez-Alvarez S, Benavente Y, Sukvirach S (2009). Geographic variation in the prevalence of Kaposi sarcoma-associated herpesvirus and risk factors for transmission.. J Infect Dis.

[4] Chen L, Lagunoff M (2005). Establishment and maintenance of Kaposi's sarcoma-associated herpesvirus latency in B cells.. J Virol.

[5] Lukac DM, Kirshner JR, Ganem D (1999). Transcriptional activation by the product of open reading frame 50 of Kaposi's sarcoma-associated herpesvirus is required for lytic viral reactivation in B cells.. J Virol.

[6] Dyson OF, Traylen CM, Akula SM (2010). Cell membrane-bound Kaposi's sarcoma-associated herpesvirus-encoded glycoprotein B promotes virus latency by regulating expression of cellular Egr-1.. J Biol Chem.

[7] Knapska E, Kaczmarek L (2004). A gene for neuronal plasticity in the mammalian brain: Zif268/Egr-1/NGFI-A/Krox-24/TIS8/ZENK?. Prog Neurobiol.

[8] Gashler A, Sukhatme VP (1995). Early growth response protein 1 (Egr-1): prototype of a zinc-finger family of transcription factors.. Prog Nucleic Acid Res Mol Biol.

[9] Thiel G, Cibelli G (2002). Regulation of life and death by the zinc finger transcription factor Egr-1.. J Cell Physiol.

[10] Renne R, Zhong W, Herndier B, McGrath M, Abbey N (1996). Lytic growth of Kaposi's sarcoma-associated herpesvirus (human herpesvirus 8) in culture.. Nat Med.

[11] McCoy C, Smith DE, Cornwell MM (1995). 12-O-tetradecanoylphorbol-13-acetate activation of the MDR1 promoter is mediated by EGR1.. Mol Cell Biol.

[12] Ford PW, Bryan BA, Dyson OF, Weidner DA, Chintalgattu V (2006). Raf/MEK/ERK signalling triggers reactivation of Kaposi's sarcoma-associated herpesvirus latency.. J Gen Virol.

[13] Cohen A, Brodie C, Sarid R (2006). An essential role of ERK signalling in TPA-induced reactivation of Kaposi's sarcoma-associated herpesvirus.. J Gen Virol.

[14] Aggarwal BB, Bhardwaj A, Aggarwal RS, Seeram NP, Shishodia S (2004). Role of resveratrol in prevention and therapy of cancer: preclinical and clinical studies.. Anticancer Res.

[15] Beck H, Semisch M, Culmsee C, Plesnila N, Hatzopoulos AK (2008). Egr-1 regulates expression of the glial scar component phosphacan in astrocytes after experimental stroke.. Am J Pathol.

[16] Cao X, Mahendran R, Guy GR, Tan YH (1993). Detection and characterization of cellular EGR-1 binding to its recognition site.. J Biol Chem.

[17] Cai Y, Liu Y, Zhang X (2006). Induction of transcription factor Egr-1 gene expression in astrocytoma cells by Murine coronavirus infection.. Virology.

[18] Romagnoli L, Sariyer IK, Tung J, Feliciano M, Sawaya BE (2008). Early growth response-1 protein is induced by JC virus infection and binds and regulates the JC virus promoter.. Virology.

[19] Katsarou K, Lavdas AA, Tsitoura P, Serti E, Markoulatos P (2010). Endocytosis of hepatitis C virus non-enveloped capsid-like particles induces MAPK-ERK1/2 signaling events.. Cell Mol Life Sci.

[20] Bedadala GR, Palem JR, Graham L, Hill JM, McFerrin HE (2011). Lytic HSV-1 infection induces the multifunctional transcription factor Early Growth Response-1 (EGR-1) in rabbit corneal cells.. Virol J.

[21] Chang Y, Moore PS (1996). Kaposi's Sarcoma (KS)-associated herpesvirus and its role in KS.. Infect Agents Dis.

[22] Glaunsinger B, Ganem D (2004). Lytic KSHV infection inhibits host gene expression by accelerating global mRNA turnover.. Mol Cell.

[23] De Leo A, Arena G, Stecca C, Raciti M, Mattia E (2011). Resveratrol inhibits proliferation and survival of Epstein Barr virus-infected Burkitt's lymphoma cells depending on viral latency program.. Mol Cancer Res.

[24] Matsumura S, Fujita Y, Gomez E, Tanese N, Wilson AC (2005). Activation of the Kaposi's sarcoma-associated herpesvirus major latency locus by the lytic switch protein RTA (ORF50).. J Virol.

[25] Fagone E, Conte E, Gili E, Fruciano M, Pistorio MP (2011). Resveratrol inhibits transforming growth factor-beta-induced proliferation and differentiation of ex vivo human lung fibroblasts into myofibroblasts through ERK/Akt inhibition and PTEN restoration.. Exp Lung Res.

[26] Lee SJ, Kim MM (2011). Resveratrol with antioxidant activity inhibits matrix metalloproteinase via modulation of SIRT1 in human fibrosarcoma cells.. Life Sci.

[27] Yiu CY, Chen SY, Chang LK, Chiu YF, Lin TP (2010). Inhibitory effects of resveratrol on the Epstein-Barr virus lytic cycle.. Molecules.

[28] Bryan BA, Dyson OF, Akula SM (2006). Identifying cellular genes crucial for the reactivation of Kaposi's sarcoma-associated herpesvirus latency.. J Gen Virol.

[29] Yu F, Harada JN, Brown HJ, Deng H, Song MJ (2007). Systematic identification of cellular signals reactivating Kaposi sarcoma-associated herpesvirus.. PLoS Pathog.

[30] Sauer L, Gitenay D, Vo C, Baron VT (2010). Mutant p53 initiates a feedback loop that involves Egr-1/EGF receptor/ERK in prostate cancer cells.. Oncogene.

[31] Cai Q, Verma SC, Choi JY, Ma M, Robertson ES (2010). Kaposi's sarcoma-associated herpesvirus inhibits interleukin-4-mediated STAT6 phosphorylation to regulate apoptosis and maintain latency.. J Virol.

[32] Brown HJ, Song MJ, Deng H, Wu TT, Cheng G (2003). NF-kappaB inhibits gammaherpesvirus lytic replication.. J Virol.

[33] Yu F, Feng J, Harada JN, Chanda SK, Kenney SC (2007). B cell terminal differentiation factor XBP-1 induces reactivation of Kaposi's sarcoma-associated herpesvirus.. FEBS Lett.

[34] Faith SA, Sweet TJ, Bailey E, Booth T, Docherty JJ (2006). Resveratrol suppresses nuclear factor-kappaB in herpes simplex virus infected cells.. Antiviral Res.

[35] Docherty JJ, Sweet TJ, Bailey E, Faith SA, Booth T (2006). Resveratrol inhibition of varicella-zoster virus replication in vitro.. Antiviral Res.

[36] Krishnan V, Zeichner SL (2004). Host cell gene expression during human immunodeficiency virus type 1 latency and reactivation and effects of targeting genes that are differentially expressed in viral latency.. J Virol.

[37] Nakamura M, Saito H, Ikeda M, Hokari R, Kato N (2010). An antioxidant resveratrol significantly enhanced replication of hepatitis C virus.. World J Gastroenterol.

[38] Shukla Y, Singh R (2011). Resveratrol and cellular mechanisms of cancer prevention.. Ann N Y Acad Sci.

[39] Hua J, Guerin KI, Chen J, Michan S, Stahl A (2011). Resveratrol inhibits pathologic retinal neovascularization in Vldlr(−/−) mice.. Invest Ophthalmol Vis Sci.

[40] Venkatesan B, Valente AJ, Reddy VS, Siwik DA, Chandrasekar B (2009). Resveratrol blocks interleukin-18-EMMPRIN cross-regulation and smooth muscle cell migration.. Am J Physiol Heart Circ Physiol.

[41] Yu HW, Liu QF, Liu GN (2010). Positive regulation of the Egr-1/osteopontin positive feedback loop in rat vascular smooth muscle cells by TGF-beta, ERK, JNK, and p38 MAPK signaling.. Biochem Biophys Res Commun.

[42] Ke J, Gururajan M, Kumar A, Simmons A, Turcios L (2006). The role of MAPKs in B cell receptor-induced down-regulation of Egr-1 in immature B lymphoma cells.. J Biol Chem.

[43] Ford PW, Hamden KE, Whitman AG, McCubrey JA, Akula SM (2004). Vascular Endothelial Growth Factor Augments Human Herpesvirus-8 (HHV-8/KSHV) Infection.. Cancer Biol Ther.

[44] Akula SM, Pramod NP, Wang FZ, Chandran B (2002). Integrin alpha3beta1 (CD 49c/29) is a cellular receptor for Kaposi's sarcoma-associated herpesvirus (KSHV/HHV-8) entry into the target cells.. Cell.

[45] Akula SM, Pramod NP, Wang FZ, Chandran B (2001). Human herpesvirus 8 envelope-associated glycoprotein B interacts with heparan sulfate-like moieties.. Virology.

[46] Dyson OF, Oxendine TL, Hamden KE, Ford PW, Akula SM (2008). Differential regulation of the attachment of Kaposi's sarcoma-associated herpesvirus (KSHV)-infected human B cells to extracellular matrix by KSHV-encoded gB and cellular alphaV integrins.. Cell Microbiol.

[47] Dyson OF, Bryan BA, Lambert PJ, Ford PW, Akula SM (2007). beta1 Integrins Mediate Tubule Formation Induced by Supernatants Derived from KSHV-Infected Cells.. Intervirology.

