# Supplementary figures and images for: Resveratrol Inhibits KSHV Reactivation by Lowering the Levels of Cellular EGR-1
Source: PLoS One. 2012 Mar 12;7(3):e33364. doi: 10.1371/journal.pone.0033364 (PMC3299779; doi:10.1371/journal.pone.0033364)

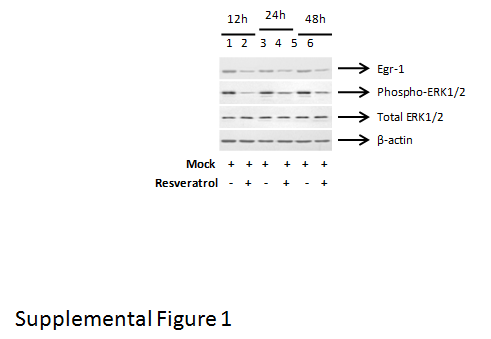

Supplement: Figure S1 — Resveratrol inhibits Egr-1 expression in the absence of KSHV infection. HEK293 cells were mock-infected by incubating with growth medium for 2 h at 37°C. These cells were washed and cultured in growth medium in the presence or absence of 100 µM of resveratrol for 48 h. The cells were lysed using gold lysis buffer (GLB) and the lysates were resolved on a 10% SDS-PAGE, transferred to a PVDF membrane, and Western blotting was performed using specific antibodies. (TIF) [file pone.0033364.s001.tif]
